# Discrete choice experiment for eliciting preference for health services for patients with ALS and their informal caregivers

**DOI:** 10.1186/s12913-021-06191-z

**Published:** 2021-03-09

**Authors:** Katy Tobin, Sinead Maguire, Bernie Corr, Charles Normand, Orla Hardiman, Miriam Galvin

**Affiliations:** 1 Global Brain Health Institute, School of Medicine, Trinity College Dublin, Dublin, Ireland; 2 Department of Neurology, National Neuroscience Centre, Beaumont Hospital, Dublin, Ireland; 3 Academic Unit of Neurology, Trinity Biomedical Sciences Institute, Trinity College Dublin, Dublin, Ireland; 4 Centre for Health Policy and Management, School of Medicine, Trinity College Dublin, Dublin, Ireland; 5 FutureNeuro SFI Research Centre, Royal College of Surgeons in Ireland, Dublin, Ireland

**Keywords:** Discrete choice experiment, Amyotrophic Lateral sclerosis, Patient preference, Caregiver preference, Health services

## Abstract

**Background:**

Amyotrophic Lateral Sclerosis (ALS) is a progressive neurodegenerative condition with a mean life expectancy of 3 years from first symptom. Understanding the factors that are important to both patients and their caregivers has the potential to enhance service delivery and engagement, and improve efficiency. The Discrete Choice Experiment (DCE) is a stated preferences method which asks service users to make trade-offs for various attributes of health services. This method is used to quantify preferences and shows the relative importance of the attributes in the experiment, to the service user.

**Methods:**

A DCE with nine choice sets was developed to measure the preferences for health services of ALS patients and their caregivers and the relative importance of various aspects of care, such as timing of care, availability of services, and decision making. The DCE was presented to patients with ALS, and their caregivers, recruited from a national multidisciplinary clinic. A random effects probit model was applied to estimate the impact of each attribute on a participant’s choice.

**Results:**

Patients demonstrated the strongest preferences about timing of receiving information about ALS. A strong preference was also placed on seeing the hospice care team later rather than early on in the illness. Patients also indicated their willingness to consider the use of communication devices. Grouping by stage of disease, patients who were in earlier stages of disease showed a strong preference for receipt of extensive information about ALS at the time of diagnosis. Caregivers showed a strong preference for engagement with healthcare professionals, an attribute that was not prioritised by patients.

**Conclusions:**

The DCE method can be useful in uncovering priorities of patients and caregivers with ALS. Patients and caregivers have different priorities relating to health services and the provision of care in ALS, and patient preferences differ based on the stage and duration of their illness. Multidisciplinary teams must calibrate the delivery of care in the context of the differing expectations, needs and priorities of the patient/caregiver dyad.

**Supplementary Information:**

The online version contains supplementary material available at 10.1186/s12913-021-06191-z.

## Background

Amyotrophic Lateral Sclerosis (ALS) is a progressive neurodegenerative condition with a mean life expectancy of 3 years from first symptom. There is no effective disease modifying treatment, and management is symptomatic and palliative.

Incorporating the needs of patients and caregivers into the evaluation and development of health services is now recognized as best practice, and most service development and health research protocols require the inclusion of the patient voice. Effective management of ALS depends not only on expertise within the clinical domain, but also on ensuring that impact on the patient, family, health and social environments has been evaluated and addressed. This is because values attributed by patients and caregivers do not always align with those attributed by healthcare professionals [1], and a full understanding of the factors that are important to the service user has the potential to enhance service delivery and engagement and improve efficiency. Additionally, recognizing that some aspects of care provision can be objectively beneficial but unattractive to users is important [2]. Such insights can help to adjust the manner by which care is delivered, taking into account the autonomy of patients and the needs of caregivers, and can also help to inform and improve communication between providers and users of services.

Understanding the perspectives and complex needs of both patients and their caregivers is often challenging for health care professionals. There may be competing benefits and limitations that are difficult to disentangle from the perspective of those providing care within a multidisciplinary clinic [3]. Various methodologies have been utilized by clinic providers to determine the views of patients and caregivers ranging from quantitative (questionnaire) based analyses, mixed methods, and qualitative studies in which patient and caregivers are interviewed and thematic analysis performed. Each methodology has strengths and weaknesses [4–7]. In ALS, qualitative research with patients and caregivers has explored needs [8–12], decision making [13, 14], experience of services [15, 16] and communication preferences [17, 18]. Effective decision-making in ALS occurs when patients’ values are supported by care providers [14]. The application of a DCE methodology in this study provides the opportunity to elicit the preferences of patients and caregivers regarding treatment and care options.

Additional mathematical based experimental paradigms can be applied to determine the perspective of service users, including the concept of the discrete choice experiment. The Discrete Choice Experiment (DCE) is a stated preferences method used frequently in economic research, which asks service users to make trade-offs for various attributes of health services. This method is used to quantify preferences and shows the relative importance of the attributes in the experiment, to the service user.

This is increasingly used in the health sector to evaluate interventions and understand the preferences of service users [19–22]. The method has been applied in palliative care to elicit preferences for the types of services available [23]. In that setting, the highest value was placed on the availability of specialist therapies, above medical support.

In a DCE, participants are asked to choose between pairs of choice profiles. The profiles are made up of several attributes which describe aspects of healthcare provision and health services, and each attribute is made up of different levels [22].

Here, we aim to use a DCE to measure the preferences for health services of ALS patients and caregivers and the relative importance of various aspects of care, such as timing of care, availability of services, and decision making. We also aim to determine whether preferences vary by sex, type of ALS onset and stage of disease to gain insights which may inform how care is delivered.

## Methods

This DCE presented participants with a series of choice sets, made up of a number of attributes and levels. Participants were asked to choose between two alternative packages of care in each choice set. This forces the participant to make trade-offs between their preferred and less preferred attribute levels. Analysis of these choices provides a measure of utility for each attribute level. The analysis can also test whether additional factors such as demographics and clinical characteristics are associated with different preferences.

### Development of attributes and levels

The attributes and levels for use in this DCE were derived using a phased approach. In the first phase, the patient journey of 100 patients with ALS were reviewed from first symptom to death. From this, 20 vignettes were developed to represent a spectrum of patient journeys.

In the second phase of development, the patient vignettes were presented to members of the clinical multidisciplinary team (MDT), palliative care clinicians and experts in healthcare management (*n* = 5) for roundtable discussion. This group met four times to determine the attributes that were relevant to patients and caregivers, as defined by the MDT and palliative care team. A set of attributes (13 for patients, 11 for caregivers) were defined for the experiment, with either two or three levels each (Table 1). The attributes and levels were randomised into choice sets using the rotation design method [24]. Choice sets were randomised into four blocks, and each participant was shown one block.

**Table 1:** Attributes and levels for patient and caregiver experiment

Attribute Description	Levels	Included in Patient DCE	Included in Caregiver DCE
Disease Information	A1. I would get all the information about motor neuron disease at the time of diagnosis A2. I would get all information about motor neuron disease when I think I will need it A3. I don’t want to know anything about motor neuron disease at any time	✓	✓
Price of additional services	B1. All services would be free B2. Extra services provided will cost 80euro per visit, for example physiotherapy or speech and language therapy visit B3. Extra services provided will cost 40euro per visit, for example physiotherapy or speech and language therapy visit	✓	✓
Arranging visits	C1. I would get regular visits from community multidisciplinary team (for e.g. physiotherapy, occupational therapist) and the public health nurse in my home C2. I would arrange multidisciplinary team (for e.g. physiotherapy, occupational therapist) and public health nurse visits as I need in my home	✓	✓
Distant to clinic	D1. I would attend Multidisciplinary team MND specialist clinic no matter how far away D2. I would just attend a local Neurology clinic	✓	✓
Waiting times	E1. I would be prepared to go to a multidisciplinary clinic with long waiting times in the clinic to see more than one professional E2. I would not be prepared to go to a multidisciplinary clinic with long waiting times in the clinic to see more than one professional	✓	✓
Place of care	F1. No matter what I would like to receive all my care at home F2. I would receive care in a hospital or a hospice as an inpatient	✓	✓
Decisions on care	G1. The doctors advise and help me when I need treatments including ventilation and stomach tube G2. The doctors advise me, and I would decide what treatments I get including ventilation and stomach tube	✓	
Personal care provision	H1. Personal care in the home is provided to me by someone who is not a relative or friend H2. Personal care in the home is provided by a relative or friend	✓	✓
Timing of hospice care	I1. I would start to see the Hospice Care team early on in my illness I2. I would start to see the Hospice Care team when something serious happens later in my illness	✓	
Communication devices	J1. I would use new communication technology including voice banking J2. I would not use new communication technology including voice banking	✓	
Availability of phone advice	K1. Phone advice is not available K2. Phone advice is available 24 h a day K3. Phone advice is available during the hours of 9 am – 5 pm Monday to Friday	✓	✓
Provision of emotional support	L1. Emotional support is not provided L2. Emotional support is provided by group meetings with other MND patients L3. Emotional support is provided from a counselor	✓	✓
Dependable Healthcare professionals	M1. I would have healthcare professionals who I can depend on M2. I would not necessarily need healthcare professionals to depend on	✓	✓
Who helps in the home	N1. I would have non-related individuals in my house to provide home help for my loved one N2. I would not have non-related individuals in my house to provide home help for my loved one		✓

The wording in this table was used for the patient DCE. In the caregiver DCE the wording was changed to reflect the caregiver’s perspective. For example in Attribute C1 the wording changed from “I would get regular visits” to “My loved one would get regular visits”

The third phase of development comprised a pilot study within which a sample of five patients and five caregivers were asked to participate in the DCE and review and provide feedback on the attributes and levels, and the number of choice sets presented to them. During the pilot, nine choice sets were presented to each participant, which was not found to be overly burdensome. Participants were asked to choose between two options, an opt-out option was not presented and each choice set had some overlapping levels. The final version of the DCE content was developed following this pilot phase. The final design remained unchanged from the pilot study (Table 1).

### Data collection and recruitment

Patients and their caregivers were recruited on consecutive weeks from a national multidisciplinary clinic as they attended scheduled appointments from April 2014 to May 2015. We aimed to recruit as many participants as possible during this study time frame to achieve a sample that would produce reliable results. Previous research has indicated that sample sizes of approximately *n* = 20 typically provide reliable estimates, but larger sample sizes are required for estimating covariate effects [25]. Patients aged 18 years or over who fulfilled the El Escorial Diagnostic Criteria for Possible, Probable or Definite ALS [26], and who were cognitively normal using the Edinburgh Cognitive and Behavioural ALS Screen (ECAS) [27], were eligible to participate. Caregivers of patients who were participating in the study who were aged 18 years or over were eligible to participate. Additional variables (e.g. sex, age, date of diagnosis and site of onset of disease) were collected from study participants and/or medical records.

Consenting participants who met the eligibility criteria were presented with a paper copy of the experiment and asked to give their preferred option from each of the scenarios or choice set presented.

Each choice set consisted of two hypothetical packages of care, and participants were asked to choose the package that they preferred. This process was repeated nine times with each participant. On average the DCE took 20 min to complete for both patients and caregivers.

### Statistical design and analysis

Design of the instrument and analysis of the data were carried out using the support. CEs package [28] in R [29]. The attribute levels were randomised into choice sets using the rotation design method [24]. The choice sets contained all of the attributes, but the levels of each attribute varied across choice sets. A random effects probit model was applied to estimate the impact of each attribute on a participant’s choice. The model estimates the coefficient for each attribute level, and the magnitude of the coefficient is related to the tendency for participants to choose that attribute level. A large positive coefficient indicates that study participants preferred that level of the attribute, while a negative coefficient indicates a negative preference for an attribute level. The magnitude of attribute coefficients and their associated 95% confidence interval were used as an indicator of the strength of participant preferences.

Additional variables were added as binary factors to examine whether different subgroups demonstrate different preferences. The variables included were sex (male or female), site of onset (bulbar or spinal onset), time since diagnosis (≤ 6 months or > 6 months), and Kings Clinical Staging (Stage 1/2 or Stage 3/4) [30]. Analysis of the patient DCE was stratified to further investigate differences between subgroups of the study sample based on sex, site of onset, King’s clinical stage and time since diagnosis.

## Results

### Patient DCE

The demographic characteristics of 93 patients and 56 caregivers are described in Table 2. Recruitment numbers for caregivers were lower than for patients either because patients attended the clinic visit alone, or caregivers chose not to participate (*n* = 19). All patients who were approached agreed to participate and were consented, and all participants were included in the analysis.

**Table 2:** Profile of study sample

Characteristics	Patients (***N*** = 93) n(%)	Caregivers (***N*** = 56) n(%)
Sex: Male	55 (59.1)	21 (37.5)
Mean age (range)	61 (25–86)	51 (28–78)
Caregiver relationship to patient:		
Spouse/partner	–	36 (64.3)
Child of patient	–	17 (30.4)
Other	–	3 (5.4)
Site of onset:		
Bulbar	23 (24.7)	–
Spinal	69 (74.1)	–
Cognitive	1 (1.1)	–
Median time in months since diagnosis (Q_1_, Q_3_)	12.2 (5.5, 30.6)	
Diagnosis </= 6 months	23 (24.7)	
Diagnosis > 6 months	70 (75.3)	
King’s Clinical Staging		
Stage 1 or 2	26 (28.0)	
Stage 3 or 4	62 (66.7)	

The results of the random effects probit analysis of the patient DCE data are outlined in Table 3. Patients showed strong preferences for three attributes (timing of information, use of hospices services and communication devices, (A, I, J)).

**Table 3:** Results of Random effects probit model for patient DCE full analysis, and subgroup analysis by time of diagnosis

Attribute	Full Analysis Coefficient (95% Confidence Interval)	Subgroup Analysis	
		Diagnosis ≤ 6 months Coefficient (95% Confidence Interval)	Diagnosis > 6 months Coefficient (95% Confidence Interval)
Intercept	-0.21 (−0.49, 0.07)	− 0.44 (−1.03, 0.13)	− 0.08 (− 0.39, 0.23)
*Reference level*			
*A1. I would get all the information about motor neuron disease at the time of diagnosis*			
A2. I would get all information about motor neuron disease when I think I will need it	**−0.22 (− 0.44, − 0.01)**	−0.21 (− 0.67, 0.24)	**−0.25 (− 0.50, − 0.01)**
A3. I don’t want to know anything about motor neuron disease at any time	− 0.18 (− 0.40, 0.05)	− 0.19 (− 0.65, 0.27)	−0.24 (− 0.50, 0.01)
B. Price of additional services	− 0.00 (− 0.00, 0.00)	0.00 (− 0.01, 0.01)	0.00 (− 0.01, 0.00)
*Reference level*			
*C1. I would get regular visits from community multidisciplinary team (for* e.g. *physiotherapy, occupational therapist) and the public health nurse in my home*			
C2. I would arrange multidisciplinary team (for e.g. physiotherapy, occupational therapist) and public health nurse visits as I need in my home	0.07 (−0.08, 0.22)	0.05 (− 0.26, 0.35)	0.02 (− 0.16, 0.19)
*Reference level*			
*D1. I would attend Multidisciplinary team MND specialist clinic no matter how far away*			
D2. I would just attend a local Neurology clinic	−0.11 (− 0.31, 0.08)	− 0.14 (− 0.55, 0.26)	−0.01 (− 0.23, 0.22)
*Reference level*			
*E1. I would be prepared to go to a multidisciplinary clinic with long waiting times in the clinic to see more than one professional*			
E2. I would not be prepared to go to a multidisciplinary clinic with long waiting times in the clinic to see more than one professional	0.04 (−0.13, 0.22)	0.27 (−0.09, 0.64)	0.00 (− 0.20, 0.20)
*Reference level*			
*F1. No matter what I would like to receive all my care at home*			
F2. I would receive care in a hospital or a hospice as an inpatient	−0.05 (− 0.20, 0.11)	−0.08 (− 0.24, 0.04)	−0.06 (− 0.24, 0.12)
*Reference level*			
*G1. The doctors advise and help me when I need treatments including ventilation and stomach tube*			
G2. The doctors advise me, and I would decide what treatments I get including ventilation and stomach tube	−0.11 (− 0.26, 0.03)	**−0.35 (− 0.67, − 0.05)**	−0.07 (− 0.23, 0.10)
*Reference level*			
*H1. Personal care in the home is provided to me by someone who is not a relative or friend*			
H2. Personal care in the home is provided by a relative or friend	−0.04 (− 0.23, 0.16)	−0.28 (− 0.73, 0.17)	0.00 (− 0.22, 0.22)
*Reference level*			
*I1. I would start to see the Hospice Care team early on in my illness*			
I2. I would start to see the Hospice Care team when something serious happens later in my illness	**0.16 (0.02, 0.30)**	0.21 (− 0.10, 0.51)	0.13 (− 0.03, 0.29)
*Reference level*			
*J1. I would use new communication technology including voice banking*			
J2. I would not use new communication technology including voice banking	**−0.14 (− 0.29, − 0.00)**	−0.01 (− 0.33, 0.30)	−0.17 (− 0.33, − 0.00)
*Reference level*			
*K1. Phone advice is not available*			
K2. Phone advice is available 24 h a day	0.04 (− 0.16, 0.23)	−0.06 (− 0.47, 0.34)	0.00 (− 0.22, 0.22)
K3. Phone advice is available during the hours of 9 am – 5 pm Monday to Friday	−0.09 (− 0.24, 0.05)	−0.05 (− 0.35, 0.24)	−0.11 (− 0.28, 0.05)
*Reference level*			
*L1. Emotional support is not provided*			
L2. Emotional support is provided by group meetings with other MND caregivers	0.04 (−0.15, 0.23)	0.03 (− 0.15, 0.20)	0.01 (− 0.21, 0.24)
L3. Emotional support is provided from a counselor	0.02 (−0.13, 0.17)	0.03 (− 0.30, 0.36)	0.03 (− 0.15, 0.20)
*Reference level*			
*M1. I would have healthcare professionals who I can depend on*			
M2. I would not necessarily need healthcare professionals to depend on	0.10 (−0.08, 0.28)	**0.42 (0.02, 0.83)**	0.09 (−0.12, 0.30)
Site of Onset	−0.07 (− 0.28, 0.13)	−0.2 (− 0.64, 0.24)	−0.09 (− 0.34, 0.16)
Time Since Diagnosis	− 0.01 (− 0.24, 0.21)	–	–
Sex	− 0.04 (− 0.24, 0.14)	0.39 (− 0.25, 0.73)	−0.12 (− 0.35, 0.09)
Kings Staging	0.13 (− 0.08, 0.34)	0.24 (− 0.25, 0.73)	0.00 (− 0.24, 0.25)
Rho-squared	0.13	0.2	0.1
Adjusted rho-squared	0.09	0.08	0.07

The magnitude of the coefficient is related to the tendency for participants to choose that attribute level. The magnitude of attribute coefficients and their associated 95% confidence interval were used as an indicator of the strength of participant preferences

Patients demonstrated the strongest preferences about timing of receiving information about ALS (attribute A). Patients strongly preferred receipt of all information about ALS at the time of diagnosis, rather than when they consider the information to be personally relevant (coefficient: -0.22, 95% CI: − 0.44, − 0.01). A strong preference was also placed on seeing the hospice care team (attribute I) later rather than early on in the illness (coefficient: 0.16, 95% CI: 0.02, 0.30). Patients also indicated their willingness to consider the use of communication devices (attribute J) (coefficient: -0.14, 95% CI: − 0.29, − 0.00).

All other attributes had small coefficients with wide confidence intervals, indicating that patients showed no strong preference for their inclusion in their healthcare package.

The model shows no significant differences in preferences for the binary factors sex, site of onset, time since diagnosis, or stage of disease. To further investigate subgroup differences, stratified analyses were carried out, dividing the sample according to these binary factors.

The stratified models for the DCE with patients are presented in supplementary Tables 1 and 2 (please see Additional file 1).

When grouped by sex, female patients demonstrate a strong preference for using communication devices (coefficient: -0.25, 95% CI: − 0.47, − 0.21). Males did not show a strong preference for any attribute. When grouped by site of onset, those with bulbar onset ALS were less likely to show any strong preferences. The subgroup of patients with spinal onset ALS showed a preference for delaying engagement with palliative/hospice services (coefficient: 0.17, 95% CI: 0.01, 0.33).

Grouping by stage of disease, patients who were in earlier stages of disease (King’s stage 1 or 2) showed a strong preference for receipt of extensive information about ALS at the time of diagnosis, rather than at a later stage (when clinically relevant)(coefficient: -0.51, 95% CI: − 0.99, − 0.05), or not receiving relevant information at any time during their illness (coefficient: -0.57, 95% CI: − 1.05, − 0.10). Those in later disease stages (King’s stage 3 or 4) did not show statistically significant preferences for any attribute.

### Caregiver DCE

Table 4 shows the results of the caregiver DCE estimated using a random effects probit model. In contrast to patients, caregivers showed a strong preference for engagement with healthcare professionals (Attribute M)(coefficient: -0.21, 95% CI: − 0.37, − 0.04), an attribute that was not prioritised by patients (Table 3). Sex was not a determining factor for caregiver response, nor was the relationship of the patient to caregiver – although these categories may have been limited by power.

**Table 4:** Results of Random effects probit model for caregiver DCE

Attribute	Coefficient	95% Confidence Interval
Intercept	− 0.09	−0.29, 0.10
*Reference level*		
*A1. I would get all the information about motor neuron disease at the time of diagnosis*		
A2. I would get all information about motor neuron disease when I think I will need it	0.03	−0.23, 0.29
A3. I don’t want to know anything about motor neuron disease at any time	−0.01	−0.27, 0.25
B. Price of additional services	−0.00	−0.00, 0.00
*Reference level*		
*C1. My loved one would get regular visits from community multidisciplinary team (for* e.g. *physiotherapy, occupational therapist) and the public health nurse at home*		
C2. My loved one would arrange multidisciplinary team (for e.g. physiotherapy, occupational therapist) and public health nurse visits as they need at home	−0.11	−0.29, 0.07
*Reference level*		
*D1. I would attend Multidisciplinary team MND specialist clinic no matter how far away*		
D2. I would just attend a local Neurology clinic	−0.09	−0.33, 0.15
*Reference level*		
*E1. I would be prepared to go to a multidisciplinary clinic with long waiting times in the clinic to see more than one professional*		
E2. I would not be prepared to go to a multidisciplinary clinic with long waiting times in the clinic to see more than one professional	0.09	−0.12, 0.30
*Reference level*		
*F1. No matter what I would like my loved one to receive all care at home*		
F2. My loved one would receive care in a hospital or a hospice as an inpatient	−0.08	− 0.27, 0.11
*Reference level*		
*H1. Personal care in the home is provided by someone who is not a relative or friend*		
H2. Personal care in the home is provided by a relative or friend	0.14	−0.09, 0.36
*Reference level*		
*K1. Phone advice is not available*		
K2. Phone advice is available 24 h a day	0.22	−0.01, 0.45
K3. Phone advice is available during the hours of 9 am – 5 pm Monday to Friday	0.11	−0.06, 0.29
*Reference level*		
*L1. Emotional support is not provided*		
L2. Emotional support is provided by group meetings with other MND caregivers	−0.20	− 0.43, 0.04
L3. Emotional support is provided from a counselor	−0.02	− 0.21, 0.16
*Reference level*		
*M1. I would have healthcare professionals who I can depend on*		
M2. I would not necessarily need healthcare professionals to depend on	**−0.21**	**−0.37, − 0.04**
*Reference level*		
*N1. I would have non-related individuals in my house to provide home help for my loved one*		
N2. I would not have non-related individuals in my house to provide home help for my loved one	−0.06	−0.23, 0.11
Sex	−0.14	−0.38, 0.09
Relationship to patient	−0.02	−0.23, 0.18
Rho-squared = 0.05		

The magnitude of the coefficient is related to the tendency for participants to choose that attribute level. The magnitude of attribute coefficients and their associated 95% confidence interval were used as an indicator of the strength of participant preferences

Subgroup analysis of the caregiver DCE data explored differences in preferences based on sex and relationship to the patient (Supplementary Table 3 in Additional file 1). Female caregivers showed a strong preference against receiving emotional support in a group with other caregivers, (coefficient: -0.40, 95% CI: − 0.72, − 0.08). Female caregivers also showed a preference for having healthcare professionals that they could depend on (coefficient: -0.28, 95% CI: − 0.50, − 0.07). This contrasted with male caregivers who did not show any strong preferences.

When grouped by relationship to the patient, those who were caregivers for a parent showed a strong preference for personal care in the home being provided by a relative or friend, rather than someone who is not a relative or friend (coefficient: 0.46, 95% CI: 0.01, 0.93), while spouses did not show any preferences. Caregivers for parents also preferred that emotional support is provided by a counsellor, rather than not at all (coefficient: 0.42, 95% CI: 0.06, 0.79), and showed a preference for having healthcare professionals that they could depend on (coefficient: -0.38, 95% CI: − 0.70, − 0.06).

## Discussion

This study aimed to explore the preferences and relative importance of attributes relating to health services for patients with ALS and their informal caregivers using a DCE. To our knowledge, this is the first time this quantitative method has been used to assess preferences in an ALS sample.

The findings demonstrate differences in preferences between those recently diagnosed, and those with a more established diagnosis. Those in early disease stages were more interested in receiving information about their illness at the time of diagnosis, whereas those at a later stage of the disease were less interested in learning everything about the condition. Although the experimental design did not permit explanation of stated preferences, the choices of those in the early stages of disease could be explained by a greater degrees of optimism about outcome, whereas those at later stages of illness had already experienced inexorable decline and increasing disability, but had time to adapt to the implications of their illness.

This is supported by previous research exploring the information seeking behaviours of ALS patients. The majority of patients and caregivers search for additional information after diagnosis [17, 18].

Our data also suggest that sensitive imparting of knowledge is appropriate at the time of diagnosis, although this must also be driven by the preferences of the patient, which is in turn informed by the stage of disease.

Despite best practice guidelines/evidence relating to the benefits of the early introduction of palliative care services from diagnosis, our data suggest that patients do not wish to engage with hospice/palliative care until later stages of the illness. This finding is consistent with previous qualitative ALS research which showed that control over accessing health services is of major importance, and participants expect to engage with services when they feel ready [31]. The findings also support the work of Foley et al., [31] which showed that patients consider hospice or palliative care as end-of-life care, even though all ALS care takes a palliative approach [32].

Patients ranked as important the provision and use of communication devices, although subgroup analysis shows this was not associated with bulbar onset of disease. As the type of communication device was not specified, this ranking may have reflected a general concern about loss of ability to communicate, rather than a specific engagement with technology. This is supported by an association between this preference and duration of illness, but not with site of onset of disease (bulbar versus spinal onset).

Our data have demonstrated a significant and important divergence between patient and caregivers with respect to priortised attributes. Caregivers consistently placed the highest value on external professional support. These attributes were not prioritised by patients. Similarly attributes that were valued by patients (timing of information about the illness, and availability of communication aids) were not prioritized by caregivers.

In general, patients tended to demonstrate preferences toward clinical aspects of their illness (obtaining information about disease progression and prognosis) while caregivers focussed on services and supports that could assist them.

Perhaps surprisingly, neither patients or caregivers prioritized access to specialist multidisciplinary clinics, although there is a strong evidence base indicating that specialist multidisciplinary care improves quality of life and clinical outcome. Additional work is required to ascertain the reasons for this, and to determine how best to ensure that patients and caregivers are provided with sufficient information to enable them make informed decisions about their preference for attending a non-specialist rather than a specialist clinic. Both patients and caregivers eschewed early engagement with palliative care/hospice services. This is consistent with other work suggesting that engagement with palliative care is perceived to be an admission by healthcare professional as an end stage intervention. Education and communication regarding the benefits of specialist multidisciplinary and the early introduction of palliative care (at the time of diagnosis) is required to ensure that patients and their caregivers can make informed choices about evidence based options for care.

This study has limitations. It was crossectional in design and does not provide any information regarding evolution of preferences by patients and caregivers. Only patients who attend the national MND clinic and were well enough to attend were available for study selection. The findings are limited to the set of attributes and levels that were selected for inclusion in the DCE. It is also important to note that subanalyses are limited by sample size and the model may have missed some strong preferences for specific subgroups, as in the subgroup with bulbar onset and caregiver relationship subgroup. Other factors such as marital status, education, income and age may have a significant influence on preferences.

## Conclusions

This study demonstrates that the DCE method can be useful in uncovering priorities of patients and caregivers with ALS that might not be identified using other research methods. Our data show that patients and caregivers have different priorities relating to health services and the provision of care in ALS, and that patient preferences differ based on the stage and duration of their illness.

The study suggests that the multidisciplinary team must calibrate the delivery of care in the context of the differing expectations, needs and priorities of the patient/caregiver dyad, and that communication as to the likely benefits of evidence based multidisciplinary and palliative care require prioritisation.

## Supplementary Information

**Additional file1: Supplementary Tables.** This file includes additional analyses of subgroup data. **Table 1.** Results of Random effects probit model for patient DCE – Gender and Site of Onset subgroup analysis. **Table 2.** Results of Random effects probit model for patient DCE – Time since diagnosis and King’s Staging subgroup analysis. **Table 3.** Results of Random effects probit model for caregiver DCE – Sex and Relationship to patient subgroup analysis.

## Data Availability

The datasets used and/or analysed during the current study are available from the corresponding author on reasonable request.

## Abbreviations

ALS: Amyotrophic Lateral Sclerosis
DCE: Discrete Choice Experiment
MDT: Multidisciplinary Team
